# Tiny Hydatidiform Mole Presenting As Pregnancy of Unknown Location

**DOI:** 10.7759/cureus.41062

**Published:** 2023-06-27

**Authors:** Mikiko Kobayashi, Yuriko Yokoi, Kyoko Tanaka, Akiko Hayashi, Satoshi Ohira

**Affiliations:** 1 Department of Pathology, Marunouchi Hospital, Matsumoto, JPN; 2 Department of Obstetrics and Gynecology, Marunouchi Hospital, Matsumoto, JPN

**Keywords:** pregnancy of unknown location, p57/kip2, hydatidiform mole, human chorionic gonadotrophin, dilation and curettage

## Abstract

Pregnancy of unknown location (PUL) is a condition in which a pregnancy test, such as elevation of serum or urine β-human chorionic gonadotrophin (hCG) level, is rendered positive; however, intrauterine or extrauterine pregnancy cannot be confirmed by transvaginal sonography (TVS). Diagnostic dilation and curettage (D&C) or laparoscopy may be performed to search for the pregnancy location. We experienced a case of PUL in which D&C was performed and histological examination revealed a tiny complete hydatidiform mole within the uterine contents. A retrospective review of the clinical course of this case, such as the evaluation of serum β-hCG levels and TVS findings, suggested that this medical entity could be explained by a tiny hydatidiform mole. In PUL, during D&C, when abnormal villi are detected, even if the lesion is tiny, a suspicion of a hydatidiform mole should be considered by the pathologists, and immunostaining and/or chromosome testing/molecular genotyping should be subsequently performed. Whether a tiny hydatidiform mole poses a risk of persistent gestational trophoblastic disease requires further study based on the accumulation of cases. D&C for PUL patients may be a useful procedure to determine such diagnoses and pick up cases.

## Introduction

Pregnancy of unknown location (PUL) is a condition in which a pregnancy test, such as elevation of serum or urine β-human chorionic gonadotrophin (hCG) level, is rendered positive; however, intrauterine or extrauterine pregnancy cannot be confirmed by transvaginal sonography (TVS). Final outcomes of PUL include spontaneous resolution of PUL, intrauterine pregnancy (viable or non-viable), and ectopic pregnancy, among others [1,2]. Close follow-up with imaging studies and serum β-hCG testing will establish the final diagnosis [1,2]. However, in cases where the final pregnancy location is unknown, diagnostic dilation and curettage (D&C) to confirm the presence of an intrauterine pregnancy or laparoscopy to search for the pregnancy location may be performed [3,4]. In the present case, since the serum β-hCG level increased progressively without identifying the pregnancy location by imaging, D&C was performed with the patient’s consent.

## Case presentation

Clinical summary

**Figure 1:**
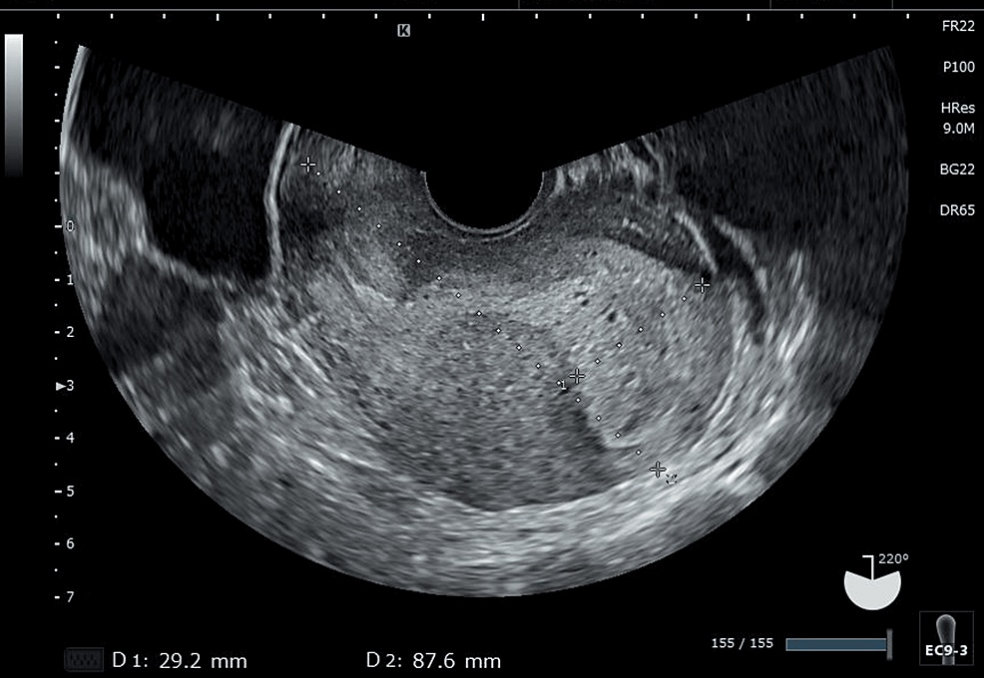
Transvaginal sonography findings of the patient at seven weeks and one day (7w1d) of gestation No gestational sac is identified in her uterus. No findings suggestive of a hydatidiform mole are also observed.

A 30-year-old healthy woman (gravida, 2; para, 1; miscarriage, 1) sought to consult with her local physician due to amenorrhea and a positive pregnancy test result from an over-the-counter pregnancy test. Her age of gestation was seven weeks and 0 days (7w0d) according to her last menstrual period. A gestational sac (GS) was absent during TVS, but her serum β-hCG level was 8,042 mIU/mL (The reference range for four to seven weeks gestation is 590-187,000 (mlU/mL).). Ectopic pregnancy was suspected. Thus, she was referred to our hospital at 7w1d of gestation. She had no symptoms and vital parameters were in the normal range. The TVS results performed during her hospital admission confirmed no GS both in the uterus (Figure 1) and in the bilateral adnexa. TVS also revealed no findings suggestive of a hydatidiform mole (Figure 1).

**Figure 2:**
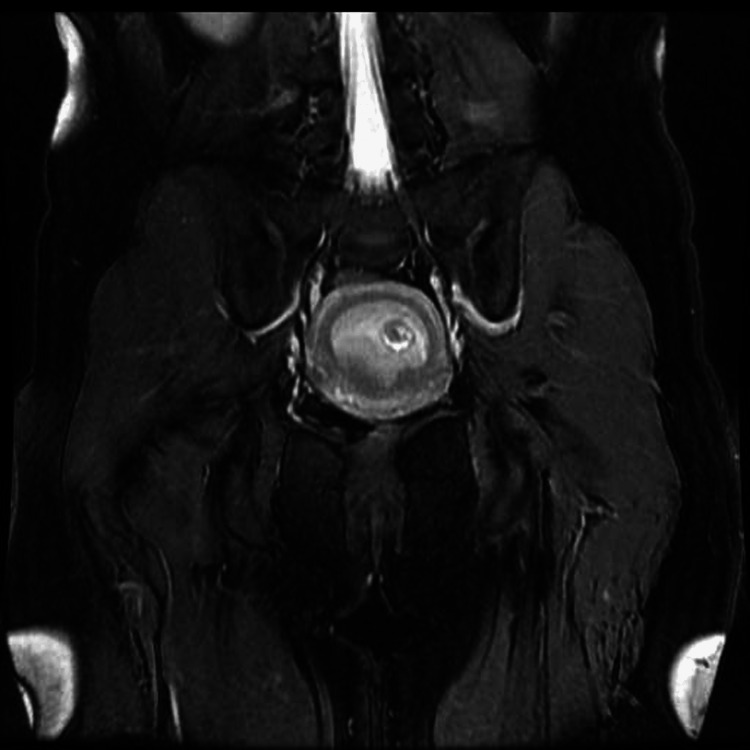
Magnetic resonance imaging of the patient at seven weeks and two days (7w2d) of gestation An intrauterine pregnancy can be suspected, but not confirmed.

 Magnetic resonance imaging could have suspected an intrauterine pregnancy but could not confirm it (Figure 2).

At 7w3d of gestation, her serum β-hCG level slightly elevated to 8,250 mIU/mL. The patient was provided the following alternatives in searching for the pregnancy location: laparoscopy or D&C. The patient selected the latter. Histological examination revealed a tiny complete hydatidiform mole within the uterine contents. Follow-up serum β-hCG level was subsequently performed. At 5.5 weeks postoperatively, the serum β-hCG level was below the cutoff value and has remained below the cutoff value until 24 weeks postoperatively.

The investigation was conducted in accordance with the Declaration of Helsinki of 1975. This case report was approved by the ethics committee of the Marunouchi Hospital, Matsumoto, Japan (No.23-3). Informed consent was obtained from the patient for the publication of patient information in the case report.

Pathological findings

**Figure 3:**
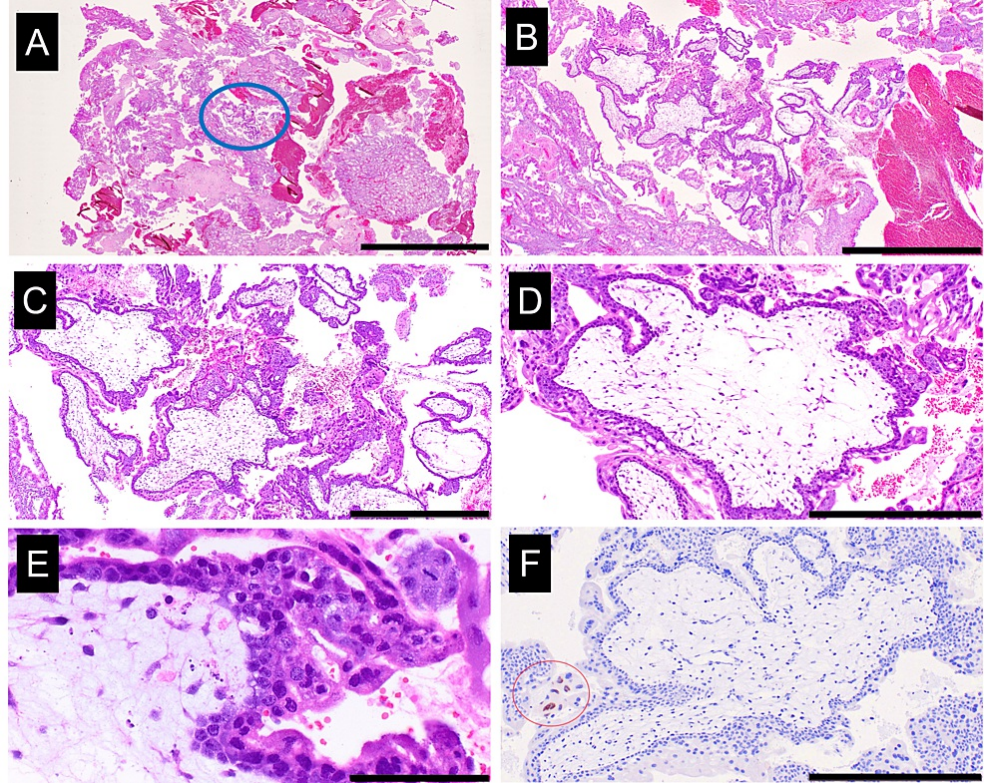
Histopathology and immunohistochemical findings of dilation and curettage specimens Hematoxylin and eosin staining (A-E) and immunostaining for p57/Kip2 (F). (A) Cluster of villi are observed in a ~3-mm diameter area (blue circle). (B, C) Some irregularly contoured villi with circumferential trophoblastic hyperplasia are observed. (D) Abnormal proliferation of trophoblasts surrounds the villi in a circumferential fashion. The villous stroma is edematous with capillary-like structures. No fetal blood cell component is observed. (E) The villous stroma contains apoptosis-like findings. (F) Immunostaining for p57/Kip2 shows positive staining for some intermediate trophoblasts (red circle) and negative for cytotrophoblasts and villous stromal cells. (A) Whole slide view, scale bar 10 mm, (B) original magnification × 40, scale bar 2 mm, (C) original magnification × 100, scale bar 1 mm, (D, F) original magnification × 200, scale bar 500 μm, and (E) original magnification × 400, scale bar 100 μm.

In curettage tissues, villi could not be identified through the naked eye. In this case, all curettage tissues were used for microscopical examination. Six formalin-fixed paraffin-embedded blocks were made. Histopathological and immunohistochemical findings of the D&C specimens are shown in Figures 3A-3E (hematoxylin and eosin staining) and Figure 3F (immunostaining for p57/Kip2). Most of the curettage tissues were decidua; however, cluster of villi were observed in a 3-mm diameter area (Figure 3A). In the area, approximately 12 villi were identified (Figure 3B). Some of these villi were irregularly contoured with circumferential trophoblastic hyperplasia (Figure 3C). Abnormal proliferation of trophoblasts surrounded the villi in a circumferential fashion (Figure 3D). The villous stroma was edematous with capillary-like structures and no fetal blood cell component was observed (Figure 3D). The villous stroma contained apoptosis-like findings (Figure 3E). Many intermediate trophoblasts were observed within the decidua. Immunostaining for p57/Kip2 showed positive staining for some intermediate trophoblasts and syncytiotrophoblasts and negative for cytotrophoblasts and villous stromal cells (Figure 3F). Based on the above findings, a diagnosis of complete hydatidiform mole was established.

## Discussion

In this case, due to the histopathological findings of morphologically abnormal villi, pathological differential diagnoses included abnormal (non-molar) villous lesions, partial hydatidiform mole, and complete hydatidiform mole, according to the World Health Organization classification of female genital tumor (5th edition) [5].

To differentiate these three conditions, morphology, immunostaining for p57/kip2, and chromosome testing/molecular genotyping are useful. Immunostaining for p57/Kip2 accurately subclassifies abnormal villous lesions into complete hydatidiform moles distinguished from the partial hydatidiform mole and non-molar specimens [6]. In complete hydatidiform mole, immunostaining for p57/Kip2 is negative in villous stromal cells and cytotrophoblasts, but positive in intermediate trophoblastic cells and maternal decidua. On the other hand, in partial hydatidiform mole and abnormal (non-molar) villous lesions, immunostaining for p57/Kip2 are positive in villous stromal cells, cytotrophoblasts, intermediate trophoblastic cells, and in the maternal decidua.

Abnormal (non-molar) villous lesions are various non-molar villous lesions with histological features simulating a partial hydatidiform mole [5], and various chromosomal or genetic alterations may be found [7]. In these conditions, p57/Kip2 is expressed in cytotrophoblasts and villous stromal cells similar to a partial hydatidiform mole [5]. These are non-molar entities and, therefore, have a low risk of persistent gestational trophoblastic disease [5]. Since most miscarriages are caused by various chromosomal abnormalities, these abnormal (non-molar) villous lesions may be seen in miscarriage specimens [5].

Initially, we considered abnormal (non-molar) villous lesions because of the tiny lesion and the fact that the imaging tests could not determine the possibility of a hydatidiform mole (nor an intrauterine pregnancy) during the course. We were uncertain whether such a tiny hydatidiform mole or a hydatidiform mole presenting as PUL, existed, although one of the causes of a missed abortion is attributed to a hydatidiform mole [8,9]. In the current case, however, immunostaining for p57/kip2 showed negative results for cytotrophoblasts and villous stromal cells. Therefore, the case was followed up as a complete hydatidiform mole. Serum β-hCG levels seemed low for a complete hydatidiform mole; however, this was thought to be attributed to its tiny size. In missed abortion, the serum β-hCG level is generally higher in the hydatidiform mole than in the non-hydatidiform mole; however, there is an overlap in the range [7]. It may be partly dependent on the volume of the lesion.

To date, only one case of hydatidiform mole presenting as PUL has been reported in Majeed’s large study regarding PUL [2]. However, this case was not reported in detail. In the current case, although the β-hCG level exceeded 8,000 mIU/mL, the location of pregnancy could not be identified on imaging, and the β-hCG level increased progressively thereafter. The initial β-hCG level and serum β-hCG ratio can predict the outcome of PUL [2-4]. A retrospective review of the clinical course of this case, such as serum β-hCG levels and TVS findings, suggested that this course could be explained by a tiny hydatidiform mole.

## Conclusions

Intrauterine pregnancy with tiny hydatidiform moles is one of the causes of PUL. D&C is a useful procedure to make this diagnosis in patients with PUL. When abnormal villi are detected in a D&C specimen, the pathologists should suspect a hydatidiform mole and perform a close examination, including immunostaining of p57/Kip2 or chromosome testing/molecular genotyping if available, even if the lesion is tiny. Whether such tiny hydatidiform mole cases have a risk of persistent gestational trophoblastic disease requires further study based on the accumulation of cases.
